# Novel Combined Tissue Transcriptome Analysis After Lentogenic Newcastle Disease Virus Challenge in Inbred Chicken Lines of Differential Resistance

**DOI:** 10.3389/fgene.2020.00011

**Published:** 2020-02-04

**Authors:** Melissa S. Deist, Rodrigo A. Gallardo, Jack C. M. Dekkers, Huaijun Zhou, Susan J. Lamont

**Affiliations:** ^1^ Department of Animal Science, Iowa State University, Ames, IA, United States; ^2^ Department of Population Health and Reproduction, School of Veterinary Medicine, University of California, Davis, Davis, CA, United States; ^3^ Department of Animal Science, University of California, Davis, Davis, CA, United States

**Keywords:** chickens, RNA-seq, weighted gene co-expression network analysis, trachea, lung, Harderian gland

## Abstract

Disease has large negative impacts on poultry production. A more comprehensive understanding of host–pathogen interaction can lead to new and improved strategies to maintain health. In particular, host genetic factors can lead to a more effective response to pathogens, hereafter termed resistance. Fayoumi and Leghorn chicken lines have demonstrated relative resistance and susceptibility, respectively, to the Newcastle disease virus (NDV) vaccine strain and many other pathogens. This biological model was used to better understand the host response to a vaccine strain of NDV across three tissues and time points, using RNA-seq. Analyzing the Harderian gland, trachea, and lung tissues together using weighted gene co-expression network analysis (WGCNA) identified important genes that were co-expressed and associated with parameters including: genetic line, days post-infection (dpi), challenge status, sex, and tissue. Pathways and driver genes, such as *EIF2AK2*, *MPEG1*, and *TNFSF13B*, associated with challenge status, dpi, and genetic line were of particular interest as candidates for disease resistance. Overall, by jointly analyzing the three tissues, this study identified genes and gene networks that led to a more comprehensive understanding of the whole animal response to lentogenic NDV than that obtained by analyzing the tissues individually.

## Introduction

Newcastle disease virus (NDV) is a single-stranded, negative-sense RNA virus. The clinical signs associated with NDV infection in chickens range from subclinical to 100% mortality depending on the virulence of the infection strain (Miller and Koch, 2013). NDV is classified into five pathotypes based on clinical signs: asymptomatic enteric, lentogenic, mesogenic, velogenic viscerotropic, and velogenic neurotropic (Afonso et al., 2012). Velogenic strains, also known in the United States as exotic NDV (END virus), have the most detrimental impacts on the poultry industry. Infection with velogenic neurotropic NDV can result in neurological clinical signs, whereas velogenic viscerotropic strains cause intestinal lesions (Afonso et al., 2012). Both velogenic strains result in high morbidity and mortality (Afonso et al., 2012). The lentogenic strains are the least virulent, and because all NDV strains belong to the same serotype, lentogenic strains are used for vaccination (Dimitrov et al., 2017). Lentogenic NDV is limited in its replication to host cells that produce a trypsin-like protease to cleave the fusion protein (Nagai et al., 1976). This restricts lentogenic NDV replication to epithelial cells where this protease is expressed (Nagai, 1995). Hence, lentogenic strains have the largest impact near sites of infection such as the trachea, lung, and Harderian gland, all of which contain epithelial cells and are also involved in mucosal immunity. These tissues, in which the virus and host have direct contact, are ideal for examining host–pathogen interaction. Increased understanding of the host–pathogen interaction creates the potential for improving strategies to curb the negative impacts of NDV.

Two inbred chicken lines have been shown to differ in their susceptibility to lentogenic La Sota NDV (Deist et al., 2017b). The Leghorn line (GHs 6) originated from a cross of two commercial lines in the 1950s and the Fayoumi line (M 15.2) originated from the Fayoum region of Egypt (Lamont and Chen, 1992). The Fayoumi and Leghorn lines are each greater than 99.95% homozygous (Fleming et al., 2016) and have different MHC types 15.2 and 6, respectively. These lines have also shown differential responses to avian influenza (Wang et al., 2014), Marek’s disease (Lakshmanan et al., 1996), *Salmonella* (Cheeseman et al., 2007), and *Eimeria* (Pinard-van der Laan et al., 2009). Inbred lines that differ in their relative susceptibility offer an excellent tool to study mechanisms of pathogen response.

The RNA sequencing (RNA-seq) approach shows the genes and pathways impacted by a treatment; however, it only shows a snapshot of response in a particular tissue and time. Because the transcriptome is not stagnant, a more comprehensive physiological picture can be obtained by an integrative analysis of the transcriptomes of multiple tissues at multiple time points after a treatment. Previously, the trachea epithelial cells, lung, and Harderian gland of Fayoumis and Leghorns at 2, 6, and 10 days post-infection (dpi) with lentogenic NDV have been individually analyzed with RNA-seq (Deist et al., 2017a; Deist et al., 2017b; Deist et al., 2018). The three tissues had very distinct responses to NDV (Deist et al., 2017a; Deist et al., 2017b; Deist et al., 2018). Taking a systems biology approach and analyzing the three tissues together will provide a more comprehensive picture of how the whole animal responded to NDV. Prior joint-tissue analyses using microarray or RNA-seq to study the impact of avian pathogenic *Escherichia coli* on broiler chickens have been performed (Sandford et al., 2012; Sun et al., 2016). Improvements in the technologies and tools for analyzing expression data now enable a more thorough examination of these complex data.

The objective of this study was to gain a more comprehensive understanding of the transcriptomic response of these two chicken lines to lentogenic NDV. Utilizing co-expression analysis across all three tissues identifies clusters of co-expressed genes that are associated with factors of interest such as NDV challenge, line, and dpi. We expect to identify novel genes and gene clusters that were not previously identified when the tissues were analyzed individually. Clusters of genes that are associated with NDV challenge and line may help identify genes potentially associated with resistance to lentogenic NDV. These data from multiple key tissues after a lentogenic challenge lay the foundation for future comparison of networks and pathways that may differ in experimental parameters including use of velogenic NDV challenge.

## Results

### Principal Component Analysis of the Trachea, Lung, and Harderian Gland

**Figure 1:**
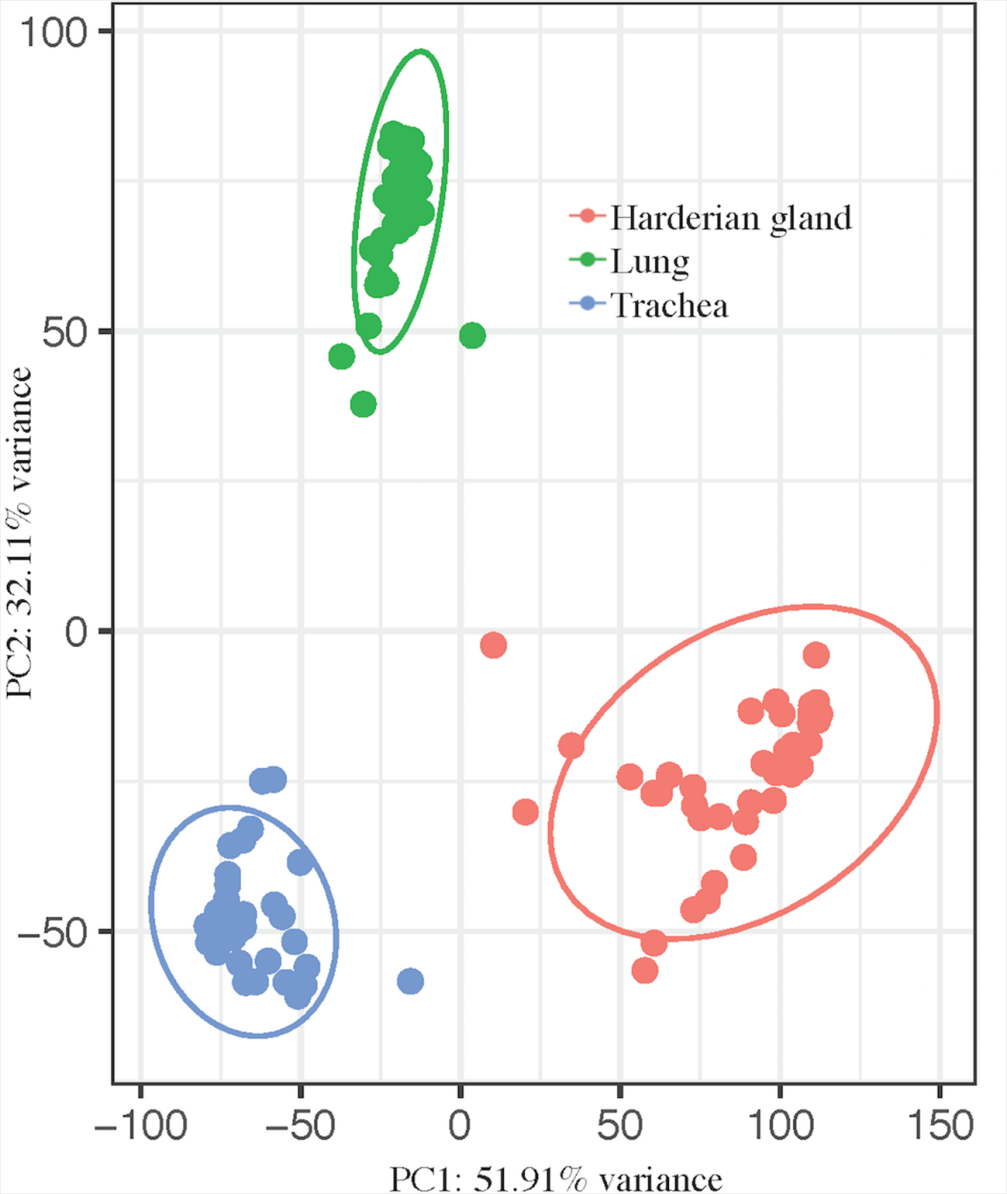
Principal component analysis shows clear separation by tissue. pcaExplorer generated PCA plot using the top 1,000 most variant transcripts. Ellipses were drawn around groups with 95% confidence interval. Groups separated by tissue, lung (green), trachea (blue), and Harderian gland (pink).

The transcriptomes of three tissues from birds infected with lentogenic NDV were jointly analyzed to more comprehensively characterize the host response to NDV. Principal component analysis showed a clear separation between the tissues, as expected (**Figure 1**). Principal component (PC) 1 (PC1 = 51.91%) separated all three tissues and PC2 (32.11%) separated the trachea and Harderian gland from the lung (**Figure 1**). No clear clustering was observed by any other parameter in all tissues.

### Differential Expression Analysis

A differential expression analysis was performed to determine if there was a difference in transcript expression levels between tissues after challenge with lentogenic NDV. The numbers of differentially expressed genes (DEG) for the challenge by tissue interaction, within each line and dpi, are shown in **Table 1**. Large numbers of DEG were identified by the interaction between the lung and Harderian gland in the Fayoumis at 10 dpi (**Table 1**), and separately the large numbers of DEG in the Leghorns at 6 dpi (**Table 1**) may have resulted from large numbers of DEG due to challenge within an individual tissue (Deist et al., 2017a; Deist et al., 2018). With the exception of the Fayoumis at 10 dpi and the Leghorns at 6 dpi, there were few DEG between the lung and Harderian gland. Interestingly, there were no DEG at 10 dpi in the Leghorns and few DEG in the Fayoumis at 6 dpi (**Table 1**). Overall, there were differences in the number of DEG from each interaction contrast, depending on genetic line and time. Of the 18 contrasts examined, *CD8B* and *LY6D* were differentially expressed most frequently in seven and six contrasts, respectively. Full lists of DEG (FDR < 0.05) for each contrast are provided in **Table S1**.

**Table 1:** Comparing the tissues’ response to lentogenic challenge within each line and time.

Line	Time	Interaction contrast^a^	DEG^b^
Fayoumi	2 dpi	Lung vs. Harderian gland	10
		Trachea vs. Harderian gland	151
		Trachea vs. lung	135
	6 dpi	Lung vs. Harderian gland	0
		Trachea vs. Harderian gland	1
		Trachea vs. lung	3
	10 dpi	Lung vs. Harderian gland	1,523
		Trachea vs. Harderian gland	54
		Trachea vs. lung	20
Leghorn	2 dpi	Lung vs. Harderian gland	0
		Trachea vs. Harderian gland	141
		Trachea vs. lung	115
	6 dpi	Lung vs. Harderian gland	743
		Trachea vs. Harderian gland	877
		Trachea vs. lung	226
	10 dpi	Lung vs. Harderian gland	0
		Trachea vs. Harderian gland	0
		Trachea vs. lung	0

a Within each line and dpi: (Tissue1, Challenged–Tissue1, Non-challenged)–(Tissue2, Challenged–Tissue2, Non-challenged).

b Differentially Expressed Genes (FDR < 0.05).

### Gene Co-Expression Analysis in the Trachea, Lung, and Harderian Gland After Lentogenic Challenge

**Figure 2:**
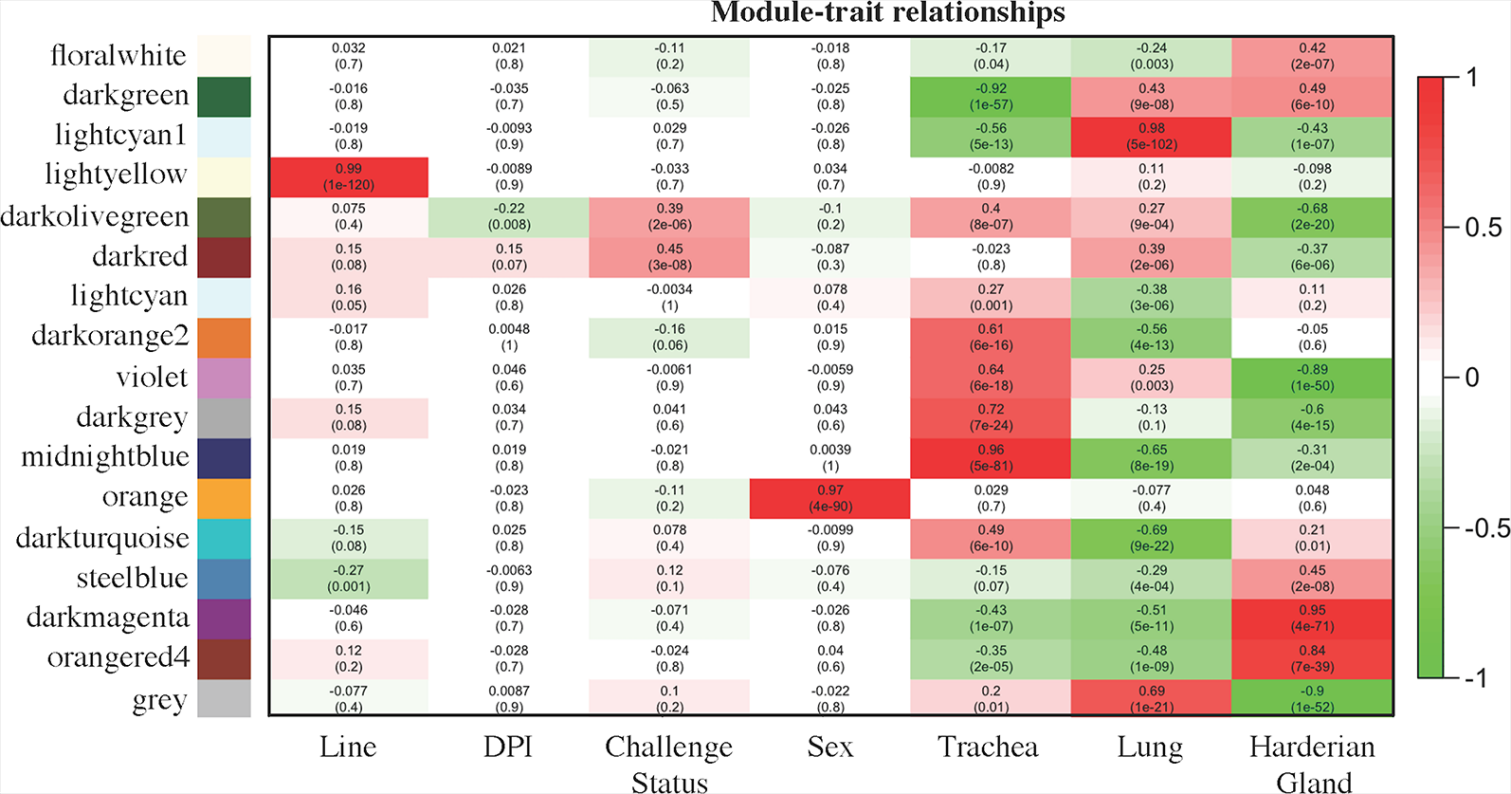
Module–trait relationships calculated by WGCNA. Factors of interest (x-axis) were correlated to each module (y-axis). The first value within each square is the correlation and the second value in parenthesis is the p value of the association. The more positively correlated the module and the factor, the more red the square; the more negatively correlated, the more green. Coding for the factors was as follows: Line [Fayoumi (1), Leghorn (0)], days post-infection [dpi; 2 dpi (0), 6 dpi (1), 10 dpi (2)], NDV [challenged (1), non-challenged (0)], sex [male (1), female (0)], Trachea [Trachea (1), Lung (0), Harderian gland (0)], Lung [Trachea (0), Lung (1), Harderian gland (0)], and Harderian gland [Trachea (0), Lung (0), Harderian gland (1)].

The module–trait relationship analysis resulted in several associations between the modules generated by weighted gene co-expression network analysis (WGCNA) based on co-expression and factors of the study: line, dpi, challenge status, sex, and tissue (**Figure 2**). The lightyellow module strongly positively correlated with line (0.99 for Fayoumi = 1 versus Leghorn = 0, *p* = 1e−120), but did not significantly correlate with any other factor, suggesting that for the 161 transcripts within this module, there was no association between line and any other factor. The lightyellow module could help better characterize the inherent differences between these two inbred genetic lines. The orange module was strongly positively correlated with sex (0.97 for male = 1 versus female = 0, *p* = 1e−90). Within this module, 94 are on the Z chromosome, 7 are on the W chromosome, and 17 transcripts have not been assigned to a chromosome. Lack of correlations with other modules indicates that these transcripts are only impacted by sex.

Three modules, midnightblue, lightcyan1, and darkmagenta, may be related to the specific functions of each tissue. The 1,308 transcripts in the midnightblue module were strongly positively correlated and more highly expressed in the trachea (0.96 for trachea = 1 versus lung and Harderian gland = 0, *p* = 5e−81). The 1,720 transcripts in the lightcyan1 module were strongly positively correlated with and more highly expressed in the lung (0.98 for lung = 1 versus trachea and Harderian gland = 0, *p* = 5e−102). The large darkmagenta module (3,154 transcripts) strongly positively correlated with and more highly expressed in the Harderian gland (0.95 for Harderian gland = 1 versus trachea and lung = 0, *p* = 4e−71).

The darkolivegreen module was negatively correlated with dpi (−0.22 for 2 dpi = 0, 6 dpi = 1, versus 10 dpi = 2, *p* = 0.0008), positively correlated with challenge status (0.39 for challenged = 1 versus non-challenged = 0, *p* = 2e−06), and negatively correlated with the Harderian gland (−0.68 for Harderian gland = 1 versus trachea and lung = 0, *p* = 2e−20). The 80 transcripts in this module were likely important for the chicken’s early response to NDV because the negative correlation with dpi indicates they were more highly expressed at early time points. The darkred module was positively correlated with challenge status (0.45 for challenged = 1 versus non-challenged = 0, *p* = 3e−08), positively correlated with the lung (0.39 for lung = 1 versus trachea and Harderian gland = 0, *p* = 2e−0.6), and negatively correlated with the Harderian gland (−0.37 for Harderian gland = 1 versus trachea and lung = 0, *p* = 6e−06). Although not significant, this module showed a weak correlation with line (0.15 for Fayoumi = 1 versus Leghorn = 0, *p* = 0.08). The darkred module potentially included genes related to response to NDV because of its associations with challenge status and line. The 333 transcripts in the steelblue module negatively correlated with line (−0.27 for Fayoumi = 1 versus Leghorn = 0, *p* = 0.001) and positively correlated with the Harderian gland (0.45 for Harderian gland = 1 versus trachea and lung = 0, *p* = 4e−71). Although not significant, this module weakly correlated with challenge status (0.12 for challenged = 1 versus non-challenged = 0, *p* = 0.1). The steelblue module may also include transcripts potentially associated with response to NDV.

Genes that are co-expressed are likely to be regulated by similar mechanisms. Within each module, the transcripts with high gene significance (GS) and module membership (MM), i.e., driver genes, were likely to have the most influence on the expression values of the entire module. The top driver genes were identified for the module that was most highly correlated with each factor (**Table 2**). Several long intervening/intergenic non-coding RNAs (lincRNAs) and unnamed protein coding genes were identified as driver genes (**Table 2**). The *NKX2-1* was among the top 10 bottom-loading genes for PC1, and *CLDN18* among the top 10 top-loading genes for PC2 calculated by pcaExplorer (Marini, 2016).

**Table 2:** Top 3 driver genes from the module most highly correlated with each factor for birds infected with lentogenic NDV.

Factor / Module	Transcript ID / Gene Name	Gene significance	Module membership
Line/lightyellow	ENSGALT00000082473/lincRNA	0.97	0.96
	ENSGALT00000065772/lincRNA	−0.97	−0.97
	ENSGALT00000087010/protein coding	0.96	0.97
DPI/darkolivegreen	ENSGALT00000017183/*EIF2AK2*	−0.37	0.83
	ENSGALT00000016499/*CMTR1*	−0.37	0.74
	ENSGALT00000066953/protein coding	−0.35	0.90
Challenge status/darkred	ENSGALT00000027228/*TNFSF13B*	0.53	0.82
	ENSGALT00000044004/*MPEG1*	0.52	0.82
	ENSGALT00000003281/protein coding	0.51	0.74
Sex/orange	ENSGALT00000077789/*Nipped-B homolog-like*	−0.98	−0.95
	ENSGALT00000047883/protein coding	−0.98	−0.96
	ENSGALT00000080994/protein coding	−0.97	−0.95
Trachea/midnightblue	ENSGALT00000021346/*akr*	0.97	0.96
	ENSGALT00000049178/*DNAL4*	0.97	0.95
	ENSGALT00000009017/*F3*	0.97	0.94
Lung/lightcyan1	ENSGALT00000089244/protein coding	0.99	0.98
	ENSGALT00000010644/*CLDN18*	0.99	0.97
	ENSGALT00000057049/lincRNA	0.98	0.97
Harderian gland/dark magenta	ENSGALT00000059647/*LRRC30*	0.98	0.98
	ENSGALT00000006028/*PABPC4*	0.97	0.94
	ENSGALT00000059800/*NKX2-1*	−0.97	−0.94

### Functional Analysis of Modules of Interest for Birds Infected With Lentogenic NDV

The darkolivegreen, darkred, and steelblue modules were of particular interest because of their correlations with either challenge status or line. The darkolivegreen module’s correlations with dpi and challenge status suggest that these genes were important in the early response to NDV. Of the 80 transcripts, 59 had an associated gene name. No modules were significantly correlated with both the challenge status and line; however, the darkred and steelblue modules may include genes associated with response to NDV because of their correlations with challenge status or line. Gene ontology (GO) term and network analysis was performed on these three modules.

**Figure 3:**
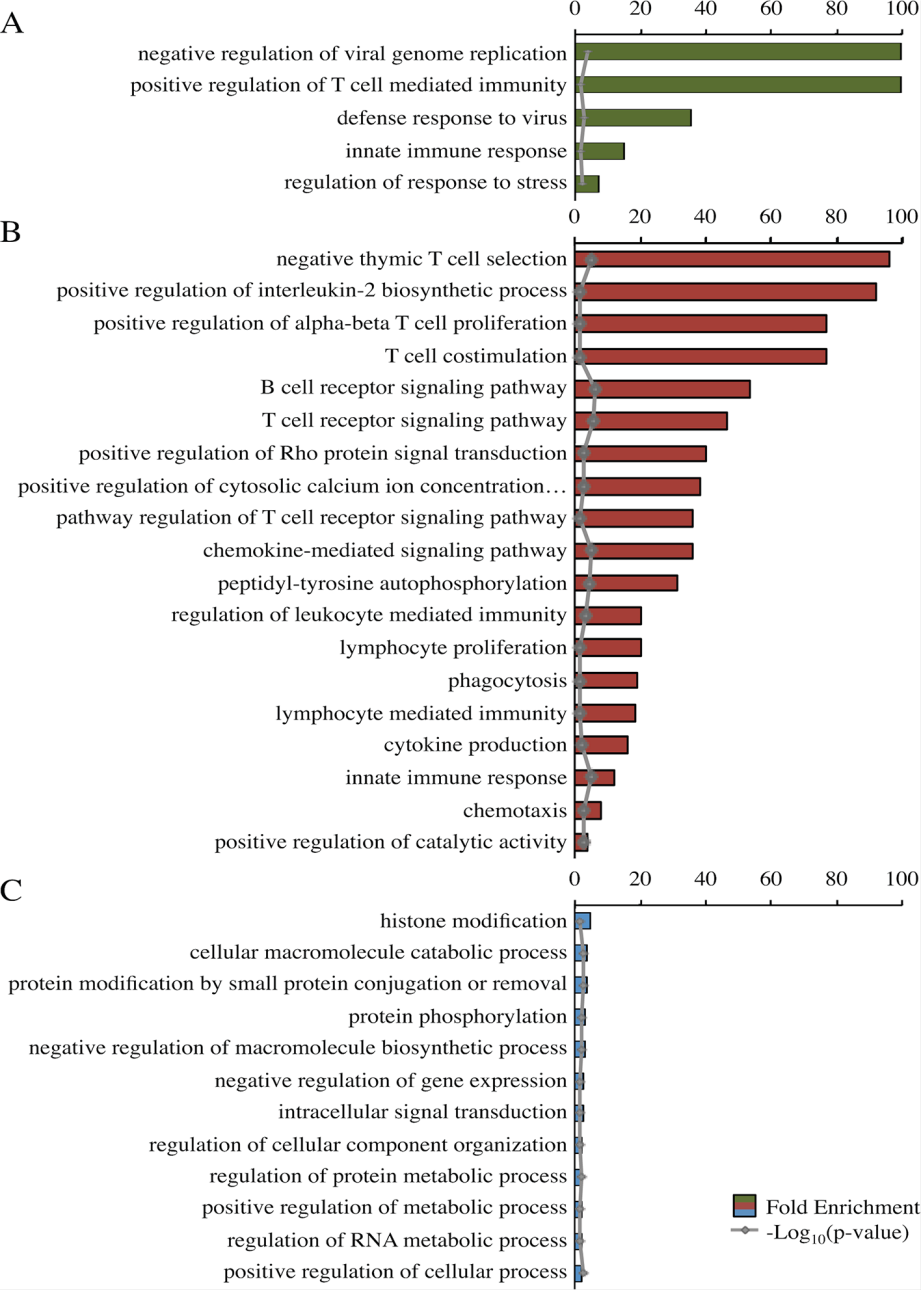
Fold enrichment of top-level overrepresented GO terms within modules of interest. Panther overrepresentation test using associated gene names for each of three modules: darkolivegreen module **(A)**, darkred module **(B)**, and steel blue module **(C)**. Fold enrichment was capped at 100. −Log10(p value) shown in gray. All p values <0.05. Ellipses indicate those involved in phospholipase C-activating G-protein-coupled signaling.

Panther identified significant top-level GO terms for the darkolivegreen, darkred, and steelblue modules (**Figure 3**). The significant GO terms for the darkolivegreen and darkred modules, both significantly correlated with challenge status, were clearly immune related. The significant GO terms for the steelblue module were more general in nature (**Figure 3**).

**Figure 4:**
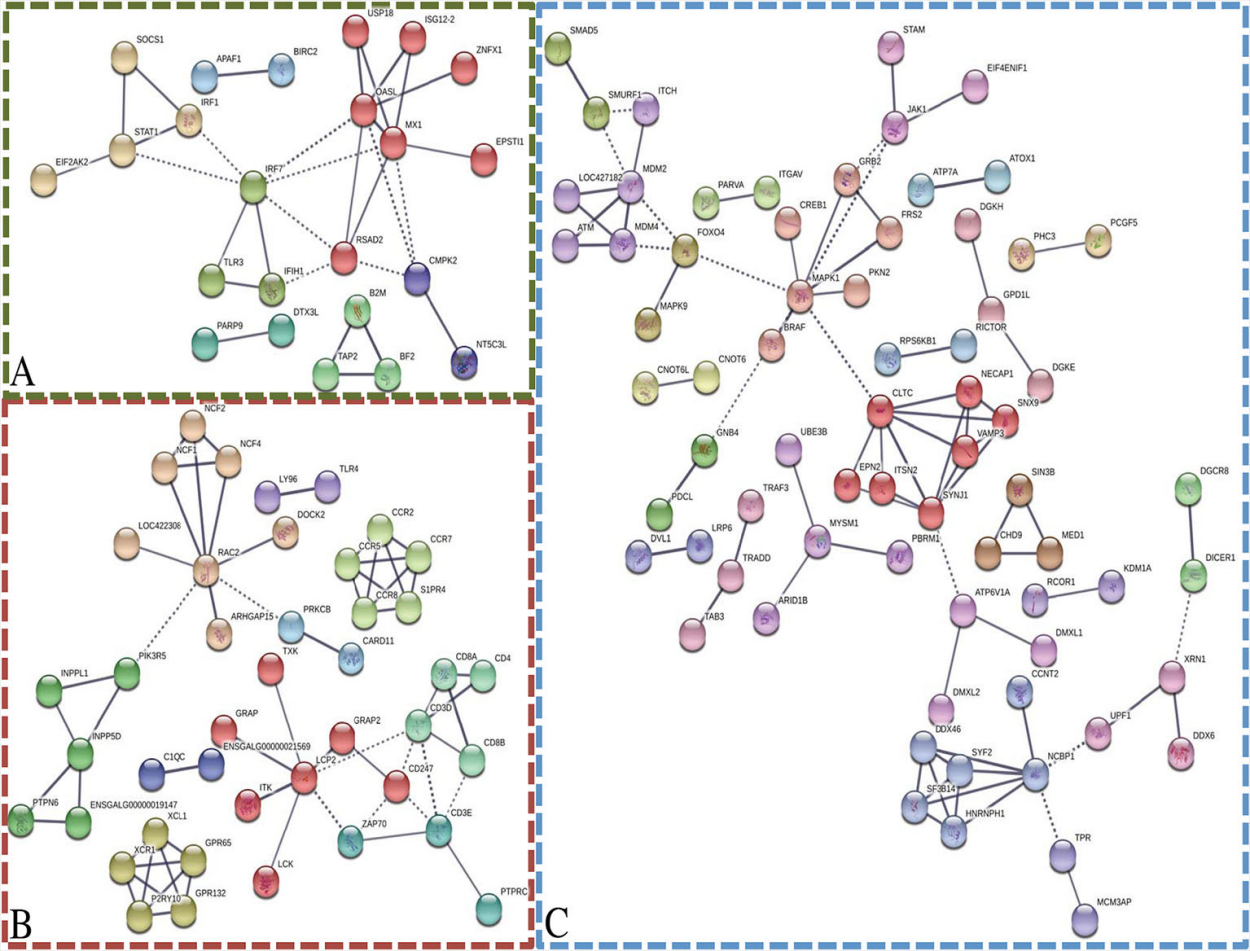
Network analysis of modules of interest. STRING was utilized to generate networks based on the associated gene name of the transcripts within the modules of interest: darkolivegreen module **(A)**, darkred module **(B)**, and steelblue module **(C)**. Disconnected nodes were removed, high confidence (0.700), and MCL clustering (inflation = 3). Solid lines indicate connections within cluster and dotted lines indicate connections between clusters.

To generate protein interaction networks for the transcripts in each module of interest, STRING was utilized (**Figure 4**). The darkolivegreen, darkred, and steelblue networks had significantly more connections than would be expected by chance. The darkolivegreen module included 55 nodes and 31 edges and was significantly associated with several KEGG pathways that are related to response to virus, including influenza A, Herpes simplex infection, and the RIG-I-like receptor signaling pathway (**Figure 4A**). Within the darkolivegreen module, *STAT1* had the second highest gene significance for challenge status and a high module membership (GS = 0.50, MM = 0.88), making the highly connected protein a potential driver gene. The darkred module network included 110 nodes and 60 edges and was significantly associated with the following KEGG pathways: cytokine–cytokine receptor interaction, cell adhesion molecules (CAMs), phagosome, Jak-STAT signaling pathway, intestinal immune network for IgA production, and phosphatidylinositol signaling system. The fourth highest driver gene, *INPP5D* (GS = 0.49, MM = 0.84), was moderately well connected (**Figure 4B**). The network generated based on the steelblue module included 251 nodes and 78 edges and was significantly associated with only one KEGG pathway: endocytosis. *DGKE* and *RICTOR* were among the top 4 driver genes in the steelblue module based on gene significance for line (**Figure 4C**).

## Discussion

The combined tissue analysis of the current study provided a comprehensive picture of the Fayoumi and Leghorn transcriptomic response to lentogenic NDV challenge in tissues where the NDV vaccine replicates. The Harderian gland, lung, and tracheal epithelial cells come into direct contact with the lentogenic virus when infected *via* the eyes and/or airways and provide the best opportunity to examine the host–pathogen interaction. Previously reported analysis of each individual tissue’s transcriptome resulted in unique findings for each tissue, as briefly summarized here. The trachea epithelial cells had the highest amount of detectable virus (Deist et al., 2017b). As time progressed, the number of DEG decreased between the challenged and non-challenged birds in the trachea, suggesting the trachea was consistently active in host response and in recovery from the virus in both lines (Deist et al., 2017b). The transcriptomes of the lung and Harderian gland were more impacted by line than by the challenge (Deist et al., 2017a; Deist et al., 2018). The Leghorn lung appeared to be non-responsive to the virus, whereas the Fayoumi lung produced a large number of DEG at 10 dpi when comparing the challenged to non-challenged birds (Deist et al., 2017a). The Harderian gland of the Leghorns showed a large number of DEG between the challenged and non-challenged birds at 6 dpi, and the Fayoumis had very few DEG at all time points (Deist et al., 2018). The challenge by tissue interaction resulted in more DEG if there were more DEG between challenged and non-challenged birds within a tissue. It is difficult to interpret the results of the challenge by tissue interaction because the tissues had time- and line-dependent responses to the virus as measured by DEG and had different amounts of detectable virus (Deist et al., 2017a; Deist et al., 2017b; Deist et al., 2018).

The complex design of the current combined, three-tissue experiment did not readily lend itself to a differential expression analysis because with three tissues, three time points, two genetic lines, and two challenge statuses, there was a very large number of possible contrasts. Using WGCNA for co-expression analysis does not rely on specific contrasts (differential expression) and can identify correlations between co-expressed genes and factors of importance in the study design. Using WGCNA to analyze multiple tissues is a novel approach that was successful. Previously, WGCNA was used to analyze the lung tissue individually (Deist et al., 2017a), which had some results in concordance with the combined tissue analysis. ENSGALT00000087010 was among the top driver genes for genetic line in the lung analysis as well as the joint analysis. In addition, *Nipped-B homolog-like* and ENSGALT00000080994 were among the top driver genes for sex in both analyses.

The darkolivegreen module was composed of genes that had a clear functional role in host defense. The unannotated transcripts within the darkolivegreen module should be explored further to identify their functions because they were co-expressed with genes important to the host response to NDV. GO term analysis of the genes in the darkolivegreen module showed they were involved in the host response to virus. *EIF2AK2* (also known as *PKR*) was the top driver gene for dpi for the darkolivegreen module. *EIF2AK2* is interferon-inducible and has important antiviral effects (Sadler and Williams, 2008). The EIF2 signaling pathway is known to inhibit NDV replication (Zhang et al., 2014). This pathway was shown to be significantly different between the two lines in both the trachea (Deist et al., 2017b) and spleen transcriptomes (Zhang et al., 2018). *EIF2AK2* phosphorylates *EIF2α*, which regulates protein synthesis (Deb et al., 2001). Also, treatment with poly(I:C) led to expression changes of *EIF2AK2* in chickens (Kim and Zhou, 2015). The negative correlation of the darkolivegreen module with dpi suggests *EIF2AK2* is likely more highly expressed at earlier times post-infection. Differential expression confirmed that the challenged birds had significantly higher *EIF2AK2* expression levels at 2 dpi when compared to 6 or 10 dpi across all tissues (data not shown). At 2 dpi, the highest levels of viral transcripts were detected in the trachea (Deist et al., 2017b) and Harderian gland (Deist et al., 2018), as measured by RNA-seq; high expression of *EIF2AK2* at 2 dpi would aid in viral clearance.

The functions of the genes in the darkred module involve both the innate and adaptive immune system, as determined by the GO term analysis and the top 2 driver genes. It is well known that TNF genes play an important role in response to pathogens. *TNFSF13B* (also known as *BAFF*) specifically aids in survival and proliferation of chicken B cells (Schneider, 2004), and was the top driver gene for challenge status in the darkred module. *TNFSF13B* receptors are expressed predominantly on chicken B cells (Schneider, 2004). One receptor for *TNFSF13B*, *TNFRSF13B*, was among the genes significant in the trachea challenge by line interaction at 6 dpi (Deist et al., 2017b) and was differentially expressed between the Fayoumi and Leghorn chickens in the lung in both non-challenged birds and birds challenged with avian influenza (Wang et al., 2014). Antibody production is required for NDV clearance (Reynolds and Maraqa, 2000; Kapczynski et al., 2013), and expression of *TNFSF13B* may critically influence B cells and their antibody production in response to NDV.

Another driver gene for challenge status in the darkred module was *MPEG1*. *MPEG1* (also known as *Perforin-2*) is expressed highly in macrophages and is an important component of the innate immune system (McCormack and Podack, 2015). The protein encoded by *MPEG1* works with complement to form membrane attack complexes and create holes in a target cell’s membrane (McCormack and Podack, 2015). *MPEG1* was 1 of 19 DEG in the Harderian gland (Deist et al., 2018) and 1 of 16 DEG in the lung (Deist et al., 2017a) between challenged and non-challenged Leghorns at 2 dpi. The infiltration of macrophages into tissues infected with NDV may result in an increase in RNA expression of MPEG1.

The two chicken lines have previously shown differences in the expression levels of *TNFSF13B* and *MPEG1* in response to viral pathogens, which may suggest a potential role in disease resistance (Deist et al., 2017b; Deist et al., 2018). *MPEG1* and *TNFSF13B*, however, were not included in the darkred network. This suggests that these proteins may have unknown or low confidence connections in STRING to the other proteins in the darkred module, or that co-expression of these genes was due to their functional similarity and that their proteins may not interact.

The steelblue module significantly correlated with chicken line and weakly correlated with challenge status. Although no immune-related GO terms were significant, network analysis revealed immune-related genes in this module, including *JAK*, *TRAF3*, *DICER1*, and *MAPK1*. This network was also associated with the endocytosis pathway, which is a mechanism by which NDV enters the host cell. It may be possible for a host to modify gene expression of this pathway to minimize viral entry. These inbred chicken lines have been used as models in multiple studies; further exploration of the lightyellow module will help to identify differences between the two lines.

The combined analysis of three target tissues generated a more extensive understanding of response to infection with lentogenic NDV at the whole organism level. Other studies have compared differences between the expression of key immune genes in these genetic lines after an NDV challenge in embryo (Schilling et al., 2018; Schilling et al., 2019) and at 3 weeks of age under heat stress conditions (Saelao et al., 2018). Also, genetic markers of interest have been identified in a commercial line (Rowland et al., 2018; Saelao et al., 2019) and African ecotypes (Walugembe et al., 2019) after infection with this same lentogenic strain. Data are being generated at a fast rate; new tools and types of analyses are needed to analyze the data in a more comprehensive way. The current study attempted to identify critical genes associated with response to NDV based on RNA-seq data from three different tissues. This new bioinformatics approach was limited by number of tissues and number of individuals per tissue. Further studies to confirm these findings are necessary. Differences between the lines demonstrated how host genetics impacts vaccine response. The darkred and steelblue modules were the modules most likely to contain genes that relate to pathogen response, and *EIF2AK2*, *MPEG1*, and *TNFSF13B* are promising candidate genes for lentogenic NDV resistance. These were not identified as candidate genes in the individual tissue analyses. Future studies are required to determine how the expression of these genes is impacted by velogenic challenge in the Fayoumi and Leghorn. This study demonstrated how WGCNA could be used to efficiently combine transcriptome data from different tissues, genetic lines, challenge statuses, and dpi to identify important gene clusters associated with factors of interest. Novel clusters and driver genes were identified in this analysis that can be used as a reference for comparison with future studies that use different NDV strains, as well as serving as a basis for investigations of host modulation of vaccine efficacy.

## Materials and Methods

This study used data from experiments approved by the Iowa State University Institutional Animal Care and Use Committee (IACUC log number 1-13-7490-G). All experiments were performed in accordance with the committee’s relevant guidelines and regulations. The experimental design for the lentogenic NDV challenge has been previously described in detail (Deist et al., 2017b). Briefly, 3-week-old chicks were either inoculated with 10^7^ EID_50_ La Sota NDV (challenged) or given PBS (non-challenged) *via* an ocular–nasal route. The relatively resistant Fayoumi and relatively susceptible Leghorn were intermixed within the challenged and non-challenged groups. Blocking for challenge status and line, at each time of 2, 6, and 10 dpi, one-third of the birds were euthanized for tissue collection. This design resulted in a total of 12 treatment groups: two challenge groups (challenged and non-challenged), two lines (Fayoumi and Leghorn), and three time points (2, 6, and 10 dpi).

Using the RNAqueous kit Total RNA Isolation Kit (Thermo Fisher Scientific, Waltham, MA, USA), RNA was isolated from the trachea epithelial cells, lungs, and Harderian glands of about four birds per treatment group. The RNA was treated with DNAse and the quality of each sample was confirmed (RQN > 8). The Illumina TruSeq RNA Library Prep Kit v2 (Illumina, San Diego, CA, USA) was used to generate cDNA libraries. Libraries were sequenced on the HiSeq2500 for 100-bp, single-end reads at the Iowa State University DNA Facility (Ames, IA, USA). The sequencing data was analyzed with a standard pipeline and the *Gallus-gallus* 5 reference genome (Gal5; GCA_000002315.3): FASTX, TopHat2 (Kim et al., 2013), and HTSeq (Anders et al., 2015). Transcripts with less than four counts across all samples were removed prior to normalization. The RNA-seq data are publicly available from ArrayExpress at EMBL-EBI under accession numbers: E-MTAB-5431, E-MTAB-5859, and E-MTAB-6038.

For data visualization of the 143 sequenced samples, pcaExplorer (Marini, 2016) was used to generate PCA plots based on count data normalized using DESeq2 (Love et al., 2014), accounting for tissue and treatment group. The variance associated with each principal component was calculated with the 1,000 most variant transcripts (Marini, 2016).

Differential expression analysis was performed to determine how the tissues responded differently to the NDV challenge using the GLM in edgeR (Robinson et al., 2010), accounting for every combination of tissue, line, dpi, and challenge status resulting in 36 levels. Within each line and dpi, the following contrasts were written to identify transcripts that were significantly impacted (FDR < 0.05) by the interaction between challenge and tissue: (Tissue i, Line j, dpi k, Challenged–Tissue i, Line j, dpi k, Non-challenged)–(Tissue i’, Line j, dpi k, Challenged–Tissue i’, Line j, dpi k, Non-challenged). With three tissues, two lines, and three time points, a total of 18 contrasts were analyzed.

Co-expression analysis was performed using WGCNA (Langfelder and Horvath, 2008). WGCNA clusters genes into modules based on expression levels and correlates a module’s eigengene to traits of interest. All transcripts with more than four counts across all samples were included. Transcript counts were normalized using the variance stabilizing transformation by DESeq2 (Love et al., 2014). The soft power threshold was set to 20 and a minimum module size of 30 was used (Langfelder and Horvath, 2008). The module’s eigengenes were correlated to factors of interest. Coding for the factors was as follows: Line [Fayoumi (1), Leghorn (0)], days post-infection [dpi; 2 dpi (0), 6 dpi (1), 10 dpi (2)], NDV [challenged (1), non-challenged (0)], sex [male (1), female (0)], Trachea [Trachea (1), Lung (0), Harderian gland (0)], Lung [Trachea (0), Lung (1), Harderian gland (0)], and Harderian gland [Trachea (0), Lung (0), Harderian gland (1)]. For each factor, the driver genes for a module were based on the transcripts with the highest absolute gene significance and module membership calculated by WGCNA (Langfelder and Horvath, 2008). Gene significance was estimated by correlating a transcript’s expression profile with a sample trait. A correlation of a module eigengene and the expression profile of each transcript was used to determine a transcript’s module membership score. All reported *p* values were taken directly from the WGCNA output.

GO term and network analyses were performed to ascertain the predicted function of specific modules. All transcript IDs were converted to their associated gene name using Ensembl BioMart, then NCBI and Uniprot were used to find gene names for those transcripts unidentified by Ensembl. Panther (Mi et al., 2017) was utilized for GO term overrepresentation analysis using a Bonferroni correction. Only the top-level GO terms, based on the hierarchical labeling by Panther, were presented (**Figure 3**). Associated gene names were used as input into STRING (Szklarczyk et al., 2017) for network analysis based on protein–protein interactions described in the literature. Disconnected nodes were removed and a high confidence (0.700) was required for connection of the nodes. The nodes were colored based on MCL clustering (inflation = 3) (Szklarczyk et al., 2017).

## Data Availability Statement

The RNA-seq data are publicly available from ArrayExpress at EMBL-EBI under accession numbers: E-MTAB-5431, E-MTAB-5859, and E-MTAB-6038.

## Ethics Statement

This study used data from experiments approved by the Iowa State University Institutional Animal Care and Use Committee (IACUC log number 1-13-7490-G). All experiments were performed in accordance with the committee’s relevant guidelines and regulations.

## Author Contributions

MD: Conceptualization of RNA-seq experiment, investigation, collected samples, isolated RNA, constructed cDNA libraries, formal analysis of RNA-seq, methodology, data visualization, writing—original draft preparation. RG: Conceptualization of experiment, experimental design, investigation—prepared viral isolate for inoculation, writing—review and editing. JD: Conceptualization of experiment, experimental design, writing—review and editing. HZ: Conceptualization of experiment, experimental design, funding acquisition, project administration, writing—review and editing. SL: Conceptualization of experiment, experimental design, provided resources (chicken genetic lines), investigation, funding acquisition, project administration, supervised analysis of data, writing—review and editing. All authors have read and approved the manuscript.

## Funding

This study was made possible by the generous support of the American people through the United States Agency for International Development (USAID) Feed the Future Innovation Lab for Genomics to Improve Poultry (cooperative agreement number AID-OAA-A-13-00080). The contents are the responsibility of the Feed the Future Innovation Lab for Genomics to Improve Poultry and do not necessarily reflect the views of USAID or the United States Government. Melissa Deist was partially supported by USDA NIFA 2013-38,420-20,496.

## Conflict of Interest

The authors declare that the research was conducted in the absence of any commercial or financial relationships that could be construed as a potential conflict of interest.

## Supplementary Material

Supplementary Table S1 The S1 Table (table 1.xlsx) includes the Ensembl transcript ID and corresponding log fold change and FDR value for all transcripts for all 18 challenge*line contrasts.
